# The Murine Th2 Locus Undergoes Epigenetic Modification in the Thymus during Fetal and Postnatal Ontogeny

**DOI:** 10.1371/journal.pone.0051587

**Published:** 2013-01-15

**Authors:** Momoko Yoshimoto, Mervin C. Yoder, Patricia Guevara, Becky Adkins

**Affiliations:** 1 Herman B. Wells Center for Pediatric Research, Department of Pediatrics, Indiana University School of Medicine, Indianapolis, Indiana, United States of America; 2 Department of Microbiology and Immunology, University of Miami Miller School of Medicine, Miami, Florida, United States of America; Michigan State University, United States of America

## Abstract

Epigenetic modifications play a central role in the differentiation and function of immune cells in adult animals. Developmentally regulated epigenetic patterns also appear to contribute to the ontogeny of the immune system. We show here that the epigenetic profile of the T-helper (Th) 2 locus undergoes changes in T lineage cells beginning in mid-gestation and extending throughout the first week of life. In particular, regulatory regions of the Th2 locus are largely methylated at CpG residues among fetal liver common lymphoid progenitor cells. The locus subsequently becomes highly hypomethylated among the downstream progeny of these cells within the fetal thymus. This hypomethylated state is preserved until birth when the locus becomes rapidly re-methylated, achieving adult-like status by 3–6 days post birth. Notably, the capacity for rapid, high level Th2 cytokine production is lost in parallel with this re-methylation. *In vitro* organ culture and *in vivo* transplantation experiments indicate that signals from the adult environment are required to achieve the postnatal methylated state. Together, these findings indicate that the Th2 bias of neonates may be conferred, in part, by an epigenetic profile inherited from fetal life. However, the fetal program is rapidly terminated post birth by the development of signals leading to the acquisition of adult-like epigenetic patterns.

## Introduction

Immune responses in human and murine neonates are often deficient in the proinflammatory Th1 arm of immunity. Typically in mice (reviewed in [1], [2], [3], [4] and often in humans [5], [6], [7], [8], this is associated with high level production of the anti-inflammatory Th2 cytokines interleukin (IL)-4 and IL-13, a state referred to as the neonatal Th2 bias. This pattern of cytokine secretion is thought to contribute to the susceptibility of young animals to infection and to the development of Th2-mediated diseases, such as allergy and asthma. Although the mechanisms underlying the robust Th2 responses of neonates are not fully understood, emerging data for both murine and human neonates have implicated epigenetic regulation in the robust expression of Th2 cytokine genes in early life.

The ability of neonatal CD4+ cells to produce high levels of Th2 cytokines is evident very early after a single stimulation *in vitro*. Freshly isolated murine neonatal CD4+ lymph node cells produce high levels of both IL-4 and IL-13 within 24 hr of activation [9]. This rapid Th2 cytokine production occurs prior to a single round of cell division. In striking contrast, it is well appreciated that adult CD4+ cells produce negligible levels of these cytokines at early time points. Instead, they require many days of Th2 lineage development, extensive proliferation, and the acquisition of multiple epigenetic modifications to produce significant levels of Th2 cytokines (reviewed in [10]. Therefore, we proposed that the epigenetic profile of the Th2 locus in naïve neonatal CD4+ cells may more closely resemble that of Th2 effectors than of naïve adult cells. Indeed, we recently found that the Th2 locus in unactivated neonatal cells has at least one pre-existing epigenetic characteristic similar to those in adult Th2 effector cells [9]. Hypomethylation of CpG residues was observed in naïve neonatal CD4+ lymph node cells and thymocytes at conserved non-coding sequence-1 (CNS-1) [9], an enhancer and co-regulator of Th2 cytokine gene expression [11], [12]. Since demethylation of CNS-1 in developing adult Th2 cells is thought to be critical for the acquisition of high level IL-4 production [13], [14], we hypothesized that the hypomethylation in neonates contributes to the rapid and robust Th2 function of neonatal CD4+ cells. In support of this idea, CNS-1 was nearly fully demethylated (85%) in early IL-4-producing neonatal CD4+ cells [9] – i.e., a level of demethylation similar to that of highly differentiated (7–8 days) adult Th2 effector cells [15]. In an important parallel between mouse and man, Vercelli and colleagues [6] recently described regions of hypomethylation at the Th2 cytokine locus in naïve cord blood CD4+ cells. Thus, pre-existing epigenetic modifications favorable for transcription at the Th2 cytokine locus may represent a mechanism for the rapid activation of Th2 cytokine gene expression in both human and murine neonates.

In the present study, we have extended our earlier findings to determine the specificity and developmental origins of the hypomethylated state of the Th2 locus in neonatal CD4+ cells. Our results show locus-specific epigenetic regulation in neonatal CD4+ cells since DNA hypomethylation is found at regulatory regions of the Th2 locus but is absent from the *Ifng* and *Foxp3* loci. The Th2 locus hypomethylation also shows lineage specificity – i.e., it is present in neonatal T cells but not B lineage cells. Interestingly, permissive histone marks do not accompany this hypomethylation, indicating that we have identified an epigenetic process occurring during ontogeny that may be selectively affecting the DNA methylation machinery. Strikingly, the neonatal hypomethylated pattern is established within the thymus early in ontogeny. While pre-thymic progenitors in the fetal liver are extensively methylated, the earliest T cell precursors within the 14 day fetal thymus are hypomethylated at the Th2 locus. The fetal/neonatal hypomethylated state is developmentally regulated post birth, with adult-like hypermethylation being acquired within the first week of life. Adoptive transfer and fetal thymus organ culture (FTOC) experiments demonstrate that environmental signals are critical for promoting the postnatal methylation of the Th2 locus in developing thymocytes. These findings indicate that we have identified a developmentally regulated epigenetic program with two distinct phases. Hypomethylation at the Th2 locus originates within the fetal thymus in mid-gestation and subsequently converts to the adult-like methylated state during early postnatal life. Importantly, the acquisition of adult-like methylation patterns is associated with the loss of the neonatal capacity for rapid and robust Th2 cytokine production. Thus, this epigenetic program has important implications for both the Th2 bias of neonatal life and the transition to adult-like function.

## Materials and Methods

### Mice

DO11.10 TCR-transgenic mice on a BALB/c background and CD45.1 or CD45.2 mice on a C57BL/6 background were bred and housed in pathogen-free conditions in the Division of Veterinary Resources at the University of Miami Miller School of Medicine or the Laboratory Animal Resource Center at Indiana University School of Medicine. For postnatal animals, female and male breeders were placed together for four days and then separated. The females from these matings were monitored from days 19 to 21 of gestation; the day of birth was called day 0 of life. For fetal animals, males and females were placed together for a single night and then separated. The day of separation was called day 0 of gestational life. All animal studies were carried out in strict accordance with the recommendations in the Guide for the Care and Use of Laboratory Animals of the National Institutes of Health. Animal protocols were approved by the University of Miami and Indiana University Animal Care and Use Committees. Surgery was performed under ketamine and xylazine anesthesia, and all efforts were made to minimize suffering.

### Antibodies and Cell Preparations for Ex Vivo Bisulfite Sequencing or Chromatin Immunoprecipitation (CHIP) Analyses

All antibodies and fluorochrome-conjugated streptavidin reagents were from BD Biosciences Pharmingen (San Diego, CA).

#### Thymocyte populations

Cell suspensions of thymocytes from female adult (6–8 weeks) mice or from mice of other indicated ages were stained with anti-CD4, anti-CD8, anti-CD44, anti-CD25, anti-TCR δ, and anti-TCR αβ. CD4 single positive (SP) (CD4^+^8^−^), DN1 (CD4^−^CD8^−^CD25^−^CD44^+^), DN3 (CD4^−^CD8^−^CD25^+^CD44^−^), total γδ+ (≥85% CD4-8-), and CD4+8-αβ+ cells were purified using a BD Aria II cell sorter. Samples routinely showed ≥99% purity.

#### B lineage cells

Neonatal preB cells were obtained by staining liver cells from 1 day old animals with anti-μ FITC and anti-CD19 PE and sorting the μ^-^CD19^+^ population [16].

#### Fetal Liver (FL) progenitors

14 day FL cells were stained with fluorescein-coupled lineage (lin) antibodies against TER119, Gr1, CD3, and B220 and with APC-conjugated anti-c-kit plus biotin-conjugated anti-IL-7R, followed by streptavidin PE. FL common lymphoid progenitor (CLP)-like cells were defined as lin^-^c-kit^int/+^IL-7R^+^ [17], [18] and sorted on a BD Aria II cell sorter.

#### Preparation of positive and negative control cells for CHIP

For positive control cells, CD4^+^ lymph node cells from 7 day old DO11.10 mice were enriched by positive selection on Miltenyi columns, per the manufacturer’s protocol. Six day Th2 effectors were prepared as previously described [9]. Briefly, 2×10^5^ CD4+ cells were plated in 96 well plates containing 0.5 µg plate-bound anti-CD3, 0.5 µg/ml anti-CD28, 10 ng/ml rIL-2, 50 ng/ml rIL-4, and 50 µg/ml anti-IFN-γ. Three days later, the cells were harvested and replated for an additional 3 days of culture in medium supplemented with rIL-2 only. For negative control cells, adult lymph node cells were stained with anti-CD4 FITC and anti-CD44-bn, followed by steptavidin PerCP. CD4^+^CD44^lo^ naïve phenotype cells were purified on a BD Aria II cell sorter.

### Isolation of Progenitors and Co-culture with OP9-DL1 Cells

E12.5 or E15.5 FL CLP-like cells were prepared as described in the preceding section. E9.5 yolk sac and E10.5 Aorta-gonad-mesonephros regions were dissected from embryos and digested with 0.125% Collagenase (Stemcell Technologies) to make single cell suspensions. Progenitor cells were cocultured with mouse GFP+OP9-DL1 stroma [19] in 24-well plates in 1.0 ml α-MEM (GIBCO/Invitrogen) containing 10% fetal bovine serum (Hyclone), 5×10^−5^ M 2-mercaptoethanol, 1% penicillin/streptomycin and supplemented with IL7 (10 ng/ml) [20]. At the indicated times, the cells were stained with a cocktail of FITC-labeled antibodies against lineage markers (B220, Mac-1, Gr-1, TER119), APC-conjugated anti-CD4 and anti-CD8, PE-conjugated anti-CD25, and PE-cy-7-conjugated anti-CD44. DN3, defined as GFP^-^lin-CD4^−^CD8^−^CD25^+^CD44^−^ cells, were then sort purified on a FACS Aria (Becton Dickinson).

### *In Vivo* Chimeras

Six to 8 week old adult female CD45.1+ mice were sublethally irradiated (575 rads) and 14 day fetal thymic lobes from CD45.2+ donors were transplanted under the kidney capsule, as detailed previously [21]. Briefly, host mice were anesthetized with ketamine HCL (80 µg/g body weight) and xylazine (40 µg/g body weight). The left kidney was exposed dorsally and a small incision was made in the capsule. Four intact thymic lobes were gently placed between the capsule and the body of the kidney using fine, curved forceps. The skin wound was closed with surgical staples. One and two weeks later, the transplanted thymi were removed and CD45.2+ donor CD4 SP thymocytes were purified on a BD Aria II cell sorter.

### FTOC

Culture of fetal thymic lobes has been previously described [22]. Briefly, individual thymic lobes from day 14 C57BL/6 embryos were placed onto 13-mm autoclaved 0.8 µ Nucleopore filters (Costar, Cambridge, MA) resting on Gelfoam #4 absorbable gelatin sponges (Upjohn, Kalamazoo, MI) in 35-mm tissue culture plates. Each plate received 2.0 ml of culture medium consisting of DMEM with high glucose (Life Technologies, Grand Island, NY) with 10% heat-inactivated FCS, 2 mM glutamine, 5×10^−5^M β-ME, 50 µg/ml l-asparagine, and 126 µg/ml l-arginine. Half of the medium was replaced with fresh medium very 3–4 days. One and two weeks after the initiation of culture, CD4 SP thymocytes were purified on a BD Aria II cell sorter.

### Cultures for Cytokine Production and ELISA

CD4 SP thymocytes were sorted as described in the section above entitled “Antibodies and cell preparations for ex vivo bisulfite sequencing or CHIP analyses”. CD4^+^ lymph node cells were prepared by positive selection on Miltenyi columns per the manufacturer’s protocol. 2×10^5^ cells were plated in 96 well plates and activated for 24 or 48 hr with 0.5 µg plate-bound anti-CD3 and 0.5 µg/ml anti-CD28. Supernatants were harvested and tested for IL-4 content using mouse-specific IL-4 ELISA kits (Pierce Chemical, Rockford, IL), according to the manufacturer’s directions.

### Bisulfite Sequencing

The detailed methods for bisulfite sequencing have been previously described [9]. The primer sequences and PCR parameters for CNS-1 and rad50 RHS7 were also described earlier [9]. Primer sequences and PCR conditions for *Il4* intron one (used for CIRE detection) were taken exactly from Tykocinski et al [23]. Primer sequences and PCR conditions for the other regions described in this paper were as follows:

#### IL-13 proximal promoter

Forward primer (5′-GATTTATTTTTTAAAGGTTAGGGG-3′), reverse primer (5′-ACTACTCTTCTTCCTCATTTTTA-3′). One cycle at 95°C for 2 minutes; 40 cycles at 95°C for 30 seconds, 58°C for 30 seconds, and 68°C for 1 minute 30 seconds; 1 cycle at 68°C for 7 minutes.

#### Interferon (IFN)-γ CNS-6

Forward primer (5′-TTTAATTTATGGGATAAATGAGTTA-3′); reverse primer (5′- AAATACTATCACCCCAATAACACATC-3′). One cycle at 95°C for 2 minutes; 40 cycles at 95°C for 30 seconds, 55°C for 30 seconds, and 68°C for 45 seconds; 1 cycle at 68°C for 7 minutes.

#### IFN-γ CNS-20

Forward primer (5′-GATAAGTAGTTTAAAGGTTATATGT-3′); reverse primer (5′-CTAAATCCCTTACTAACCTACATCC-3′). One cycle at 95°C for 2 minutes; 40 cycles at 95°C for 30 seconds, 51.9°C for 30 seconds, and 68°C for 45 seconds; 1 cycle at 68°C for 7 minutes.

#### FOXp3 Amplicon 1

Forward primer (5′- AGGAAGAGAAGGGGGTAGATA-3′); reverse primer (5′- AAACTAACATTCCAAAACCAAC-3′). One cycle at 95°C for 2 minutes; 40 cycles at 95°C for 1 minute, 60°C for 45 seconds, and 68°C for 1 minute; 1 cycle at 68°C for 10 minutes.

### CHIP

Chromatin immunoprecipitation was essentially performed as described by Makar et al. [15]. Briefly, sort-purified CD4+8- thymocytes were fixed in formaldehyde, solubilized, and the nuclei were centrifuged. After extraction in 1% SDS, the DNA was sheared using a Microson Ultrasonic Cell Disruptor (Model XL2000, Misonix, Inc.) with a setting of 16, 10 cycles of 10 seconds on followed by 30–60 seconds off. Agarose gel electrophoresis showed that all samples had a similar size distribution, with a main population of approximately 200–1,000 bp. Specific precipitation of 1×10^6^ cells was achieved using anti-acetyl-histone H3 and anti-dimethyl-histone H3 (lys4) (both antibodies from Upstate Biotechnology) and protein G sepharose 4 Fast Flow (GE Healthcare Bio-Sciences, Piscataway, NJ). Normal rabbit IgG (Upstate) was used for pre-clearing and as non-specific antibody control. The amount of precipitated DNA was quantified by real time PCR using an Applied Biosystems 7300 Real-Time Polymerase Chain thermocycler (Foster City, CA). CNS-1 primers were: forward (5′- CAGTTGATCTGGGAAAGTTCGTT-3′) and reverse (5′- CGGCTCCACCCAAAAGC-3′). Each 50 µl PCR reaction contained 50 nM forward primer, 50 nM reverse primer, the precipitated equivalent of 2.5×10^5^ cells, and 25 µl of Power SYBR Green PCR Master Mix (Applied Biosystems). PCR conditions were: One cycle at 50°C for 2 minutes; 1 cycle at 95°C for 10 minutes; 40 cycles at 95°C for 15 seconds, and 60°C for 1 minute. The specific precipitation value was calculated with the following equation: [DNA_nonspecificIP_] - [DNA_specificIP_]/[DNA_input_], where [DNA_specificIP_] was the amount of DNA immunoprecipitated with the antibody of interest, [DNA_nonspecificIP_] was the amount of DNA precipitated by the non-specific control antibody, and [DNA_input_] was a defined sample of sheared chromatin before immunoprecipitation.

### Data Analysis

The overall % unmethylated within each population (group of DNA clones) was determined as follows: the % of unmethylated CpG in each clone was calculated and the average among all clones within that group was determined. These average values ± the standard error of the mean are presented in graph form or above the methylation grids. Because the methylation data are non-parametric, significance was calculated using the Mann Whitney test. One-way ANOVA analyses were used for the CHIP data.

## Results

### Hypomethylation in Neonatal CD4+ Thymocytes Shows Locus and Lineage Specificity

**Figure 1:**
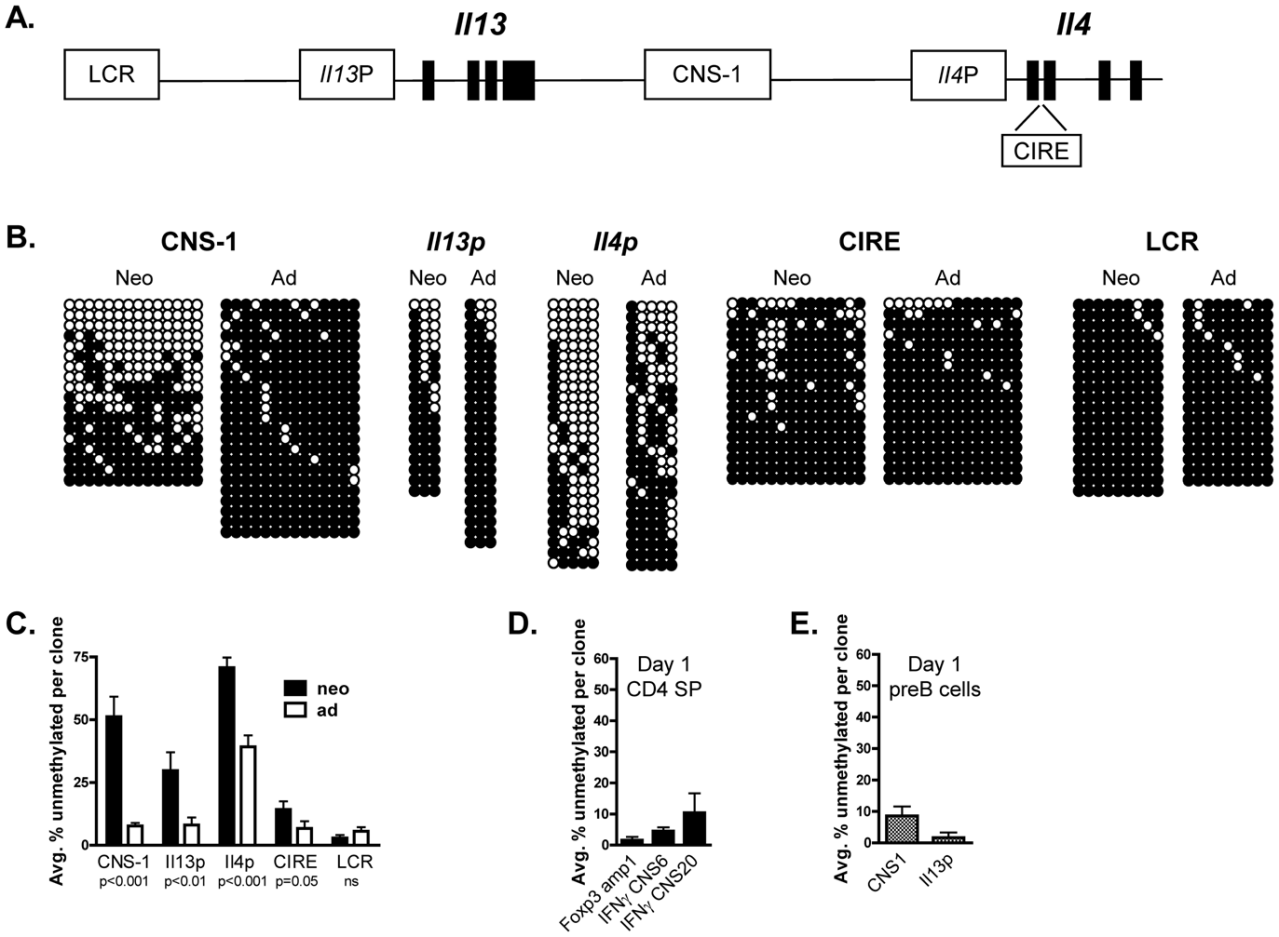
Hypomethylation at Th2 locus regulatory sites in neonatal CD4+ single positive thymocytes. (A) Schematic representation of the Th2 locus, showing the domains analyzed. (B) CD4 SP thymocytes were sort-purified from 1 day old neonatal and adult DO11.10 mice and their DNA was subject to bisulfite modification, PCR amplification, cloning, and sequencing, as described in Materials and Methods. Methylation at individual CpG residues in sites within the Th2 locus among DNA clones from two independent biological replicates is shown. ○ = unmethylated; • = methylated. (C) The data in (B) presented in graph form showing the average % ± SEM of unmethylated CpG in each group; this was determined by calculating the percentage of unmethylated CpG in each individual clone within a group and then determining the average among all clones in the group. Significance was calculated using the Mann Whitney test. (D) Bisulfite analyses of day 1 CD4 SP thymocytes for the Foxp3 amp1, IFN-γ CNS6 and IFN-γ CNS20 sequences. (E) Bisulfite analyses of Th2 locus CNS-1 and IL-13p in 1 day old neonatal μ^-^CD19^+^ preB cells.

In addition to CNS-1, other regulatory sites in the Th2 locus become hypomethylated during the development of adult Th2 effectors. Therefore, we compared the IL-13 proximal promoter (IL-13P) [24], the conserved intronic regulatory element (CIRE) in the first exon of the *Il4* gene [23], and the Th2 locus control region (LCR) [24], in addition to CNS-1, in CD4 SP thymocytes from 1 day old and adult animals (Figure 1A). As previously reported for 2 day old CD4 SP thymocytes [9], CNS -1 was substantially unmethylated in 1 day old neonatal CD4+ cells compared to adult cells (Figure 1B and C). The IL-13 promoter also showed relative hypomethylation in neonates, especially at CpG residue numbers two and three (Figure 1B and C). Strikingly, this pattern of hypomethylation is very similar to that reported by Flavell and colleagues [24] among adult CD4+ cells after 5 days of Th2 differentiation. As previously reported by Makar et al. [15], the IL-4 promoter in adult CD4 SP thymocytes was not as extensively methylated as other regions of the Th2 locus. Nonetheless, the IL-4 promoter in neonatal CD4 SP cells showed significantly greater hypomethylation (Figure 1B and C). CIRE was also significantly less methylated in neonates relative to adults (Figure 1B and C), although the differences were modest and can be largely accounted for by demethylation concentrated at only a few CpG sites (positions 5, 6, 13, and 14). Notably, the LCR, which becomes rapidly demethylated in differentiating adult Th2 effectors [24], was fully methylated in 1 day old CD4 SP thymocytes (Figure 1B and C). In addition, we previously reported [9] that a region of no known regulatory function spanning intron 1 and exon 2 of the *Il4* gene is similarly hypermethylated in neonates and adults. Therefore, hypomethylation at the neonatal Th2 locus is specific for selected regulatory regions.

Epigenetic remodeling occurs at numerous immune effector loci in developing adult effector cells. For example, upon Th1 differentiation, extensive demethylation occurs at sites distal to the *Ifng* gene and these elements are capable of enhancing IFN-γ production [25]. Similarly, conserved sequences which possess transcriptional activity and are upstream of the *Foxp3* locus become hypomethylated in natural regulatory T cells produced *in vivo* and in TGF-β induced regulatory T cells *in vitro* [26]. We utilized this information to test whether hypomethylation extended beyond the Th2 locus to other immune effector loci in neonatal life. The methylation status of two *Ifng* associated regions and one *Foxp3* site was assessed in CD4 SP thymocytes from 1 day old animals (Figure 1D). All examined sites showed extensive methylation, similar to that reported for adult naïve CD4+ cells [25], [26]. Therefore, among the gene loci we have analyzed, hypomethylation was selective for the Th2 cytokine locus.

To date, our studies have focused on CD4+ cells. It was possible that regions of the Th2 cytokine locus were hypomethylated throughout the immune system in early life. To investigate this, we prepared CD19+sIgM- pre-B cells from 1 day old neonatal liver and examined methylation at CNS-1 and the IL-13P. In contrast to the hypomethylation seen in neonatal CD4+ thymocytes, the CNS-1 and IL-13P regions in neonatal B lineage cells showed nearly complete methylation (Figure 1E).

### Histone Modifications Favorable for Transcription are Absent from the Neonatal Th2 Locus

**Figure 2:**
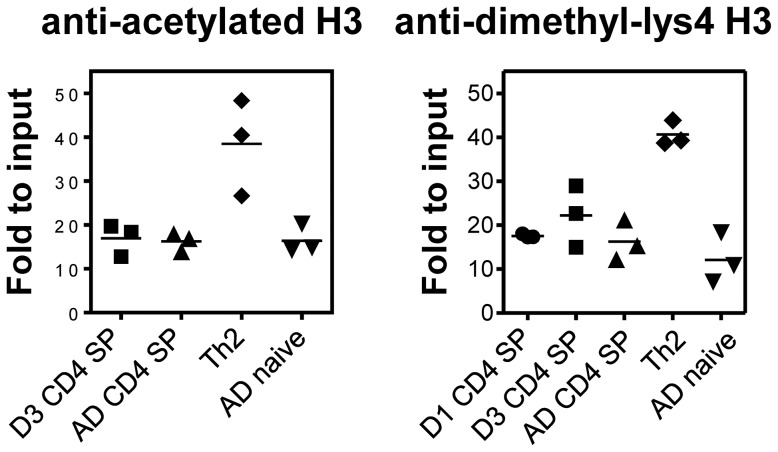
Lack of permissive histone marks at CNS-1 in neonatal thymocytes. Chromatin were prepared from sorted day 1, day 3, or adult CD4 SP thymocytes, from neonatal Th2 effectors, or from adult naïve CD4+ lymph node cells and subjected to chromatin immunoprecipitation using anti-acetylated H3 (left panel) or anti-H3 dimethyl-lys4 (right panel), followed by real-time PCR for CNS-1, as described in Materials and Methods. The horizontal lines are the mean values obtained from 3 independent experiments. Each symbol represents a different experiment.

During the development of adult Th2 effectors, modifications of histones favorable for transcription precede DNA demethylation [27]. Since regions of the Th2 locus in naïve neonatal CD4+ cells were already hypomethylated, we hypothesized that the locus would also contain favorable histone modifications. We focused on CNS-1 since histones associated with this region acquire favorable acetylation [28] and dimethylation [15] marks during Th2 differentiation. CHIP of day 1 or day 3 and adult CD4 SP thymocytes, as well as neonatal Th2 cells as the positive control and naïve CD44^lo^CD4+ adult lymph node cells as the negative control, were performed with anti-acetylated H3 and anti-H3 dimethyl-Lys4 antibodies. The precipitated material was measured by quantitative PCR (see Materials and Methods). As expected, CNS-1 was readily precipitated from neonatal Th2 cells with both antibodies (Figure 2) while naïve adult lymph node cells showed little CNS-1 recovery with the antibodies. CNS-1 was poorly precipitated with both antibodies in adult CD4+ SP thymocytes and, surprisingly, also in neonatal thymocytes. One-way ANOVA analyses indicated no significant differences with either antibody among day 1, day 3, and adult CD4 SP and adult naïve lymph node cells. Therefore, although critical regulatory domains of the Th2 locus pre-exist in a Th2 effector-like hypomethylated state in neonatal CD4+ SP thymocytes, they do not appear to harbor histone modifications favorable for transcription.

### Hypomethylation of the Th2 Locus is Present in the Early Fetal Thymus but Upstream Progenitors in the Fetal Liver are Highly Methylated

**Figure 3:**
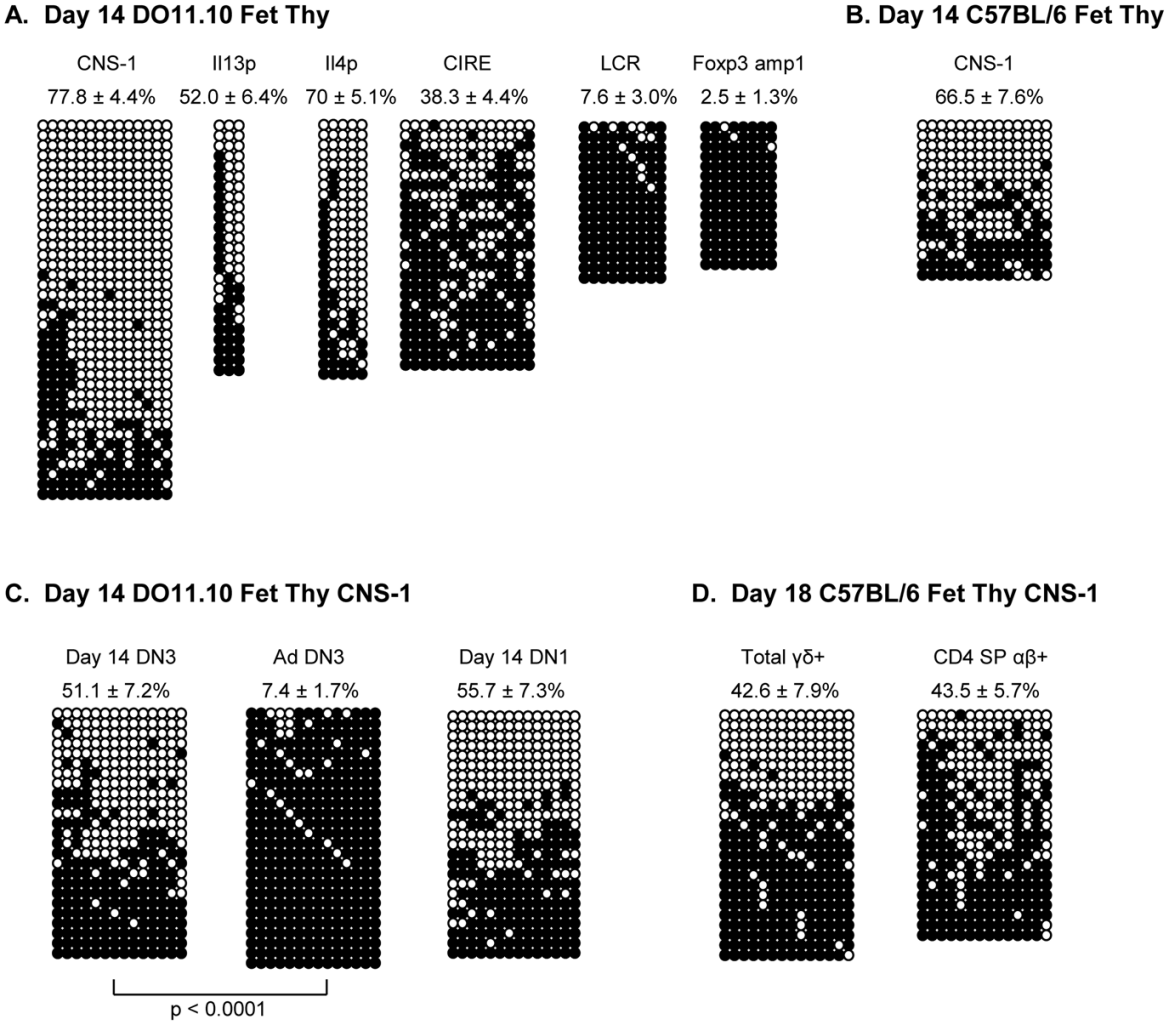
Extensive hypomethylation at the Th2 locus in day 14 fetal thymocytes. (A) Total day 14 fetal thymocytes from DO11.10 animals were assessed by bisulfite sequencing for the methylation status of CNS-1, IL-13p, IL-4p, CIRE, LCR, and Foxp3 amp1 as described for Figure 1. The average percentage unmethylated (among all of the individual clones in each group) ± SEM is indicated for each site. (B) Bisulfite sequencing analysis of CNS-1 in total day 14 fetal thymocytes from C57BL/6 animals. (C) Bisulfite sequencing analyses of CNS-1 in DN3 (CD4^−^8^−^25^+^44^−^) or DN1 (CD4^−^8^−^25^−^44^+^) cells from day 14 fetal or adult thymus, as indicated. (D) Bisulfite sequencing analyses of sort-purified γδ+ or CD4 SP αβ+ cells from 18 day fetal thymuses. All data shown are from two independent biological replicates. Mann Whitney analysis showed a significant difference (p<0.001) in methylation at CNS-1 in fetal and adult DN3 cells.

The vast majority of thymocytes present in the thymus 1 day post birth are progeny of fetal thymic precursors. Therefore, we proposed that the hypomethylation of selected Th2 locus regions may be established during gestation, within the fetal thymus. This was tested by examining the methylation status of CNS-1, IL-13P, IL-4P, and CIRE in total 14 day fetal thymocytes (Figure 3A). All four of these sequences showed extensive hypomethylation, exceeding that seen in the neonatal CD4 SP thymocyte population. Importantly, fetal thymic hypomethylation appears to be universal among mouse strains since extensive hypomethylation at CNS-1 was also observed in 14 day fetal thymocytes from C57BL/6 mice (Figure 3B). Moreover, the specificity of hypomethylation in neonatal life was also present among fetal thymocytes since the Th2 locus LCR region and *Foxp3* amplicon 1 were hypermethylated (Figure 3A). Therefore, the overall pattern and fidelity of hypomethylation observed in neonatal T cells is established as early as 14 days of gestation within the thymus.

Although all thymocytes at day 14 of ontogeny are double negative, the population is heterogeneous [29] and some of the cells are not yet fully committed to the T cell lineage. Therefore, we examined the double negative (DN) 3 subset of cells, the population at which commitment to the T cell lineage occurs (Figure 3C). CNS-1 was substantially hypomethylated in fetal DN3 cells, in contrast to the nearly fully methylated state of adult DN3 cells (Figure 3C). Hypomethylation was also observed among fetal DN1 cells, the earliest cells to enter the fetal thymus (Figure 3C). Interestingly, this hypomethylated state was retained among downstream progeny of both the αβ and γδ lineages (Figure 3D). Together, these results indicate that hypomethylation of the Th2 locus exists as far back in ontogeny as the earliest stage of fetal thymic precursors and is subsequently represented in both the αβ and γδ lineages of fetal thymocytes.

**Figure 4:**
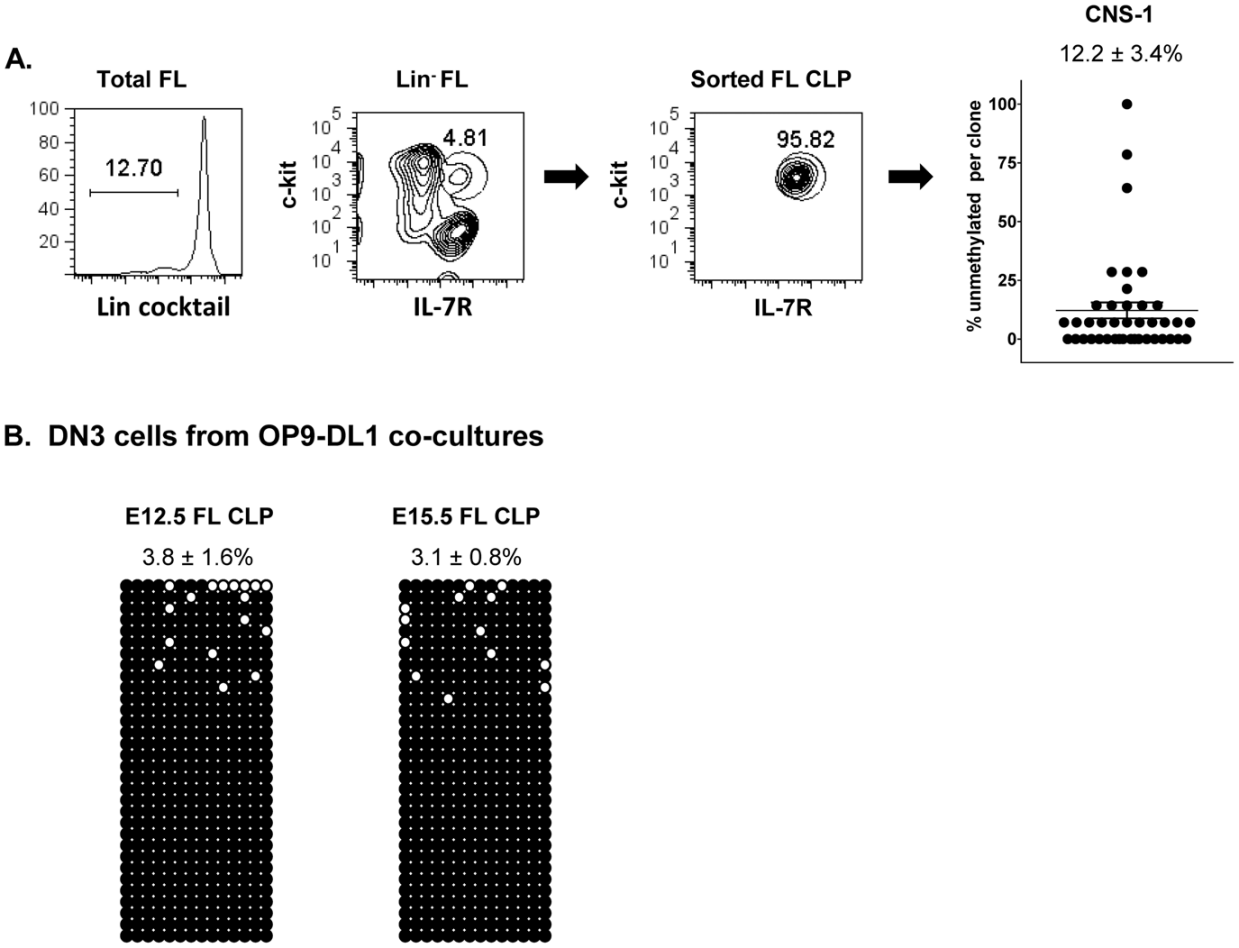
Fetal liver CLP-like cells and their progeny from OP9-DL1 cultures show Th2 locus hypermethylation. (A) CLP-like cells were isolated from C57BL/6 14 day fetal liver cells by sorting for lin^-^IL-7R^+^c-kit^+^ cells and bisulfite sequencing analysis of CNS-1 was performed. Graphed are the conglomerate data from a total of 41 clones from 3 different cellular isolates. (B) DN3 progeny from OP9-DL1 cultures of: E 12.5 FL CLP-like cells (left panel): E 15.5 FL CLP-like cells (right panel). The data are derived from three independent CLP purifications and two culture set-ups.

Hypomethylation in fetal thymic DN1 cells could arise in two possible ways: either upstream progenitors pre-exist in a hypomethylated state or demethylation occurs very rapidly upon thymic entry. To distinguish between these possibilities, we prepared FL CLP-like cells, the stage immediately upstream of the fetal thymus. Day 14 FL CLP-like cells, defined as lineage (CD3, CD11b, Gr-1, Ter119) negative, IL-7R+, c-kit+, were purified (Figure 4A) and the CNS-1 region was analyzed by bisulfite sequencing. Unlike fetal thymic DN1 cells, CNS-1 in FL CLP showed extensive methylation (compare with Figure 3D). Moreover, CNS-1 remained highly methylated in the DN3 progeny of FL CLP maturing *in vitro* in co-cultures with OP9 stromal cells expressing the delta-like Notch ligand (OP9-DL1) [19], [30] (Figure 4B). Similar analyses using DN3 progeny derived from other potential sources of fetal T cell progenitors, including yolk sac and aorta gonad mesonephros cells, showed comparable results (4.4±1.3% and 1.9±1.1% unmethylated, respectively) Together, these results indicate that the Th2 locus is methylated in pre-thymic progenitors and may require the fetal thymic microenvironment to promote its demethylation.

### Adult-like Methylation Patterns at the Th2 Locus and Adult-like Th2 Cytokine Production are Rapidly Established Post Birth

**Figure 5:**
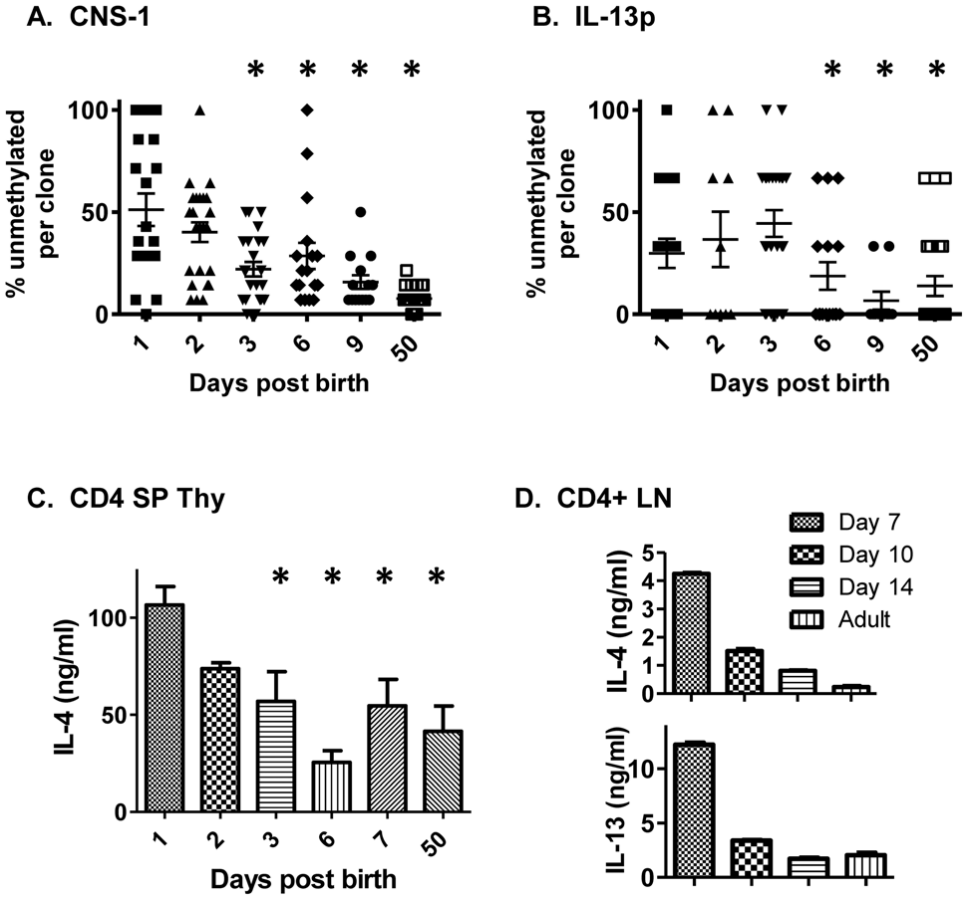
Rapid acquisition of adult-like methylation patterns post birth; association with loss of rapid IL-4 production. Sorted CD4 SP thymocytes were prepared from animals at the indicated days post birth and analyzed by bisulfite sequencing for the methylation status of CNS-1 (A) and IL-13p (B). Data from two independent cellular isolates are shown. Each symbol represents the percentage unmethylated within an individual clone; the averages among all clones are indicated by the horizontal bars. * p≤0.04 by Mann Whitney analyses, relative to day 1; all other comparisons to day 1 are not significant. (C) CD4 SP thymocytes were sort-purified and (D) CD4+ lymph node (LN) cells were prepared by positive selection on Miltenyi columns. Cells were activated for 24 (CD4 SP) or 48 (CD4+ LN) hr with plate bound anti-CD3 and anti-CD28. Supernatants were harvested and tested for IL-4 or IL-13 content using specific ELISA. *p<0.01 (Students’ t test) compared with day 1 CD4 SP.

The observation that Th2 locus regulatory regions were hypomethylated in early postnatal, but not in adult, CD4 SP thymocytes [9] (Figure 1) prompted us to examine the developmental ontogeny of this phenomenon. CD4 SP thymocytes were prepared from days 1, 2, 3, 6, and 9 post birth and from adult animals and the methylation status of the Th2 locus CNS-1 and IL-13P regions were assessed. CNS-1 became significantly methylated as early as 3 days post birth (Figure 5A) whereas the IL-13p became methylated between 3 and 6 postnatal days (Figure 5B). By 9 days, adult-like patterns of methylation were observed at both sites. Following polyclonal activation, rapid production of high levels of IL-4 was observed 1 and 2 days post birth among CD4 SP cells in the thymus (Figure 5C). Correspondingly, IL-4 and IL-13 production among CD4+ lymph node cells was evident 7 days post birth but declined shortly thereafter (Figure 5D). Therefore, hypomethylation of the Th2 cytokine locus and the capacity for rapid, high level Th2 cytokine production by CD4+ cells appear to be confined to the early postnatal period.

### The Postnatal Methylation of the Th2 Locus Requires Signals from the Environment

**Figure 6:**
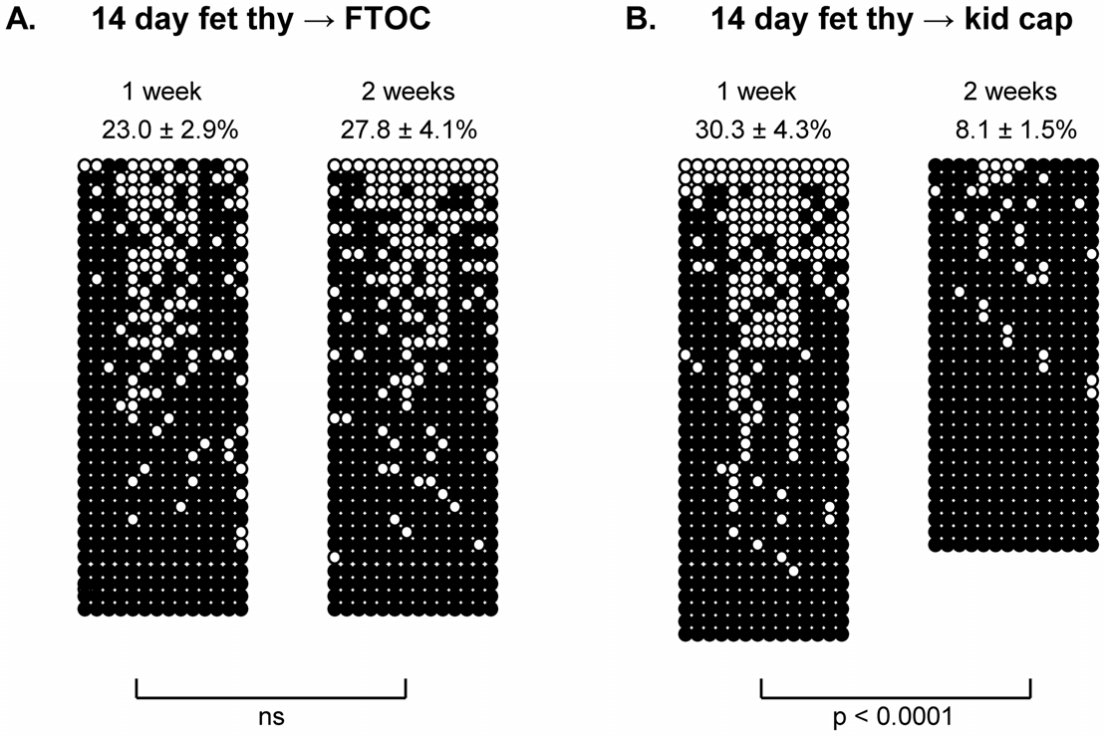
The postnatal acquisition of adult-like methylation at the Th2 locus is mediated by environmental signals. (A) Intact 14 day CD47BL/6 fetal thymi were cultured in FTOC. At the indicated times, CD4 SP thymocytes were sorted and the methylation status of CNS-1 was determined by bisulfite sequencing. (B) 14 day fetal thymic lobes were transplanted under the kidney capsule of CD45-congenic adult host mice. At the indicated times, the fetal thymi were resected and donor CD4 SP thymocytes were sorted. Methylation at CNS-1 was measured by bisulfite sequencing. All data are from 2 individual biological isolates. Mann Whitney analyses were performed, as indicated.

The transition from the fetal hypomethylated state to an adult-like hypermethylated state at the Th2 locus could be regulated in several possible ways. For example, fetal thymic precursors may be intrinsically programmed to upregulate methylation at this locus at a defined time in ontogeny. Alternatively, external signals from the maturing environment may promote the methylation post birth. We took two independent approaches to distinguish between these possibilities. First, we separated the fetal thymus from the developing environment in situ by culturing intact 14 day fetal thymi *in vitro*. One and 2 weeks later, CD4 SP thymocytes were isolated and the methylation status of CNS-1 was assessed. Of note, 1 week of culture is equivalent in elapsed time to 1 day post birth in situ. Although the extent of hypomethylation appeared somewhat reduced in 1 week FTOC-derived CD4 SP (Figure 6A) compared to CD4 SP isolated ex vivo from 1 day old C57BL/6 mice (34.5±4.9%), this reduction was not statistically significant. The demethylation seen in the 1 week FTOC was retained out to 2 weeks of culture (Figure 6A). At the equivalent period of elapsed time in situ (1 week post birth), CNS-1 showed an adult-like hypermethylated pattern (Figure 5A). Thus, the fetal thymus appears to lack an intrinsic ability to develop adult-like hypermethylation at the Th2 locus.

In the second approach, we also removed the fetal thymi but, this time, subsequent development occurred within a mature adult environment *in vivo*. 14 day fetal thymi from CD45.2+ fetuses were transplanted under the kidney capsules of sublethally irradiated CD45.1 adult hosts. One and 2 weeks later, the transplanted thymi were resected, CD45.2+ CD4 SP were sort purified, and methylation of CNS-1 was examined. One week post transplantation, CNS-1 showed hypomethylation (Figure 6B) similar to that seen in FTOC (Figure 6A). This hypomethylated state was maintained in the transplanted thymi despite ≥150 fold greater proliferation *in vivo* relative to FTOC (Table 1). Strikingly, however, CNS-1 became highly methylated by 2 weeks of transplantation (figure 6B). Indeed, this high degree of methylation was comparable to that observed in intact animals by 1 week post birth (Figure 5A). Notably, the transplanted thymuses displayed normal phenotypic differentiation – at 2 weeks post transplantation, the thymuses contained 11.0% CD4 SP, 84.9% DP, 3.0% CD8 SP,and 1.3% DN cells. These findings imply that mature environmental signals promote methylation at the Th2 locus during postnatal life.

**Table 1:** Cell yields following FTOC or kidney capsule transplantation of 14 day fetal thymus.

	1 wk yield/lobe	2 wk yield/lobe
**FTOC**		
Exp 1	2.6×10^5^	1.8×10^5^
Exp 2	1.9×10^5^	1.0×10^5^
**KID CAP**		
Exp 1	4.4×10^7^	1.7×10^8^
Exp 2	4.2×10^7^	1.3×10^8^

## Discussion

We demonstrate here that the epigenetic status of the Th2 locus in T-lineage cells oscillates at discrete stages of ontogeny between states repressive versus permissive for gene expression. During fetal development, regulatory regions in the Th2 locus are hypermethylated among pre-thymic progenitor cells found in the fetal liver. However, these regions become rapidly demethylated within the fetal thymus as hypomethylation is observed in the DN1 population of fetal thymocytes, i.e., within the population that contains the first cells to populate the fetal thymus. Importantly, the Th2 locus is also hypomethylated within the T cell-committed DN3 population of fetal thymocytes. The hypomethylation is passed down to progeny in the fetal thymus and is maintained among thymocytes and peripheral CD4+ cells into early neonatal life. This hypomethylated state is strongly linked to rapid, high level Th2 cytokine production by murine neonatal cells. Thus, neonatal cells are epigenetically poised to develop the Th2 dominant responses seen in early life. The locus subsequently rapidly acquires the adult hypermethylated profile, achieving adult-like methylation patterns and adult-like low Th2 cytokine production within the first week of life. In adults, naïve CD4+ cells retain this hypermethylated state until activated to differentiate to the Th2 lineage. During this maturation, these same regulatory regions undergo demethylation and the locus becomes stably hypomethylated in Th2 effector cells [13], [14], [15]. Thus, the methylation patterns of the Th2 locus are dependent both on the stage of ontogeny and on the differentiation state of mature CD4+ cells.

One compelling question that emerges from these findings is “what regulates the acquisition of the adult hypermethylated state at the Th2 locus post birth?”. This specific question has important general implications since a recent genome-wide study in humans demonstrated dynamic changes in DNA methylation in CD4+ cells during the first year of life [31]. In our experiments studying the Th2 locus, methylation did not occur in isolated fetal thymic lobes over a 2 week period (Figure 6A) – i.e., the same amount of time as that elapsing *in vivo* between day 14 of gestation and 1 week post birth. Therefore, fetal thymocytes do not appear to have an intrinsic capacity to achieve methylation at the Th2 locus. However, it is clear that these same cells are competent to methylate the locus when placed in a mature adult environment for the same period of time (Figure 6B). Together, these findings suggest that the early postnatal methylation at the Th2 locus is induced by signals from the maturing environment.

An equally compelling question that arises is “what regulates the initial demethylation of the Th2 locus during fetal ontogeny?”. At this point, our knowledge is limited to settings or signals that are insufficient to promote demethylation. For example, fetal liver progenitors remain methylated at the Th2 locus when differentiated in OP9DL1 cultures (Figure 4). Moreover, the T cell progeny of fetal liver cells retain their methylated status even when cultured in 2-deoxyguanosine treated fetal thymic lobes in FTOC (6.3±2.4% unmethylated). The latter result suggests that fetal thymic epithelium, by itself, cannot promote demethylation at the fetal Th2 locus. In addition to the inducing cell type, the molecular nature of the signal(s) driving demethylation within the fetal thymus is currently unknown. In adult CD4+ cells undergoing Th2 differentiation, signals through the IL-4 receptor appear to be critical for demethylation of the Th2 locus [32], [33]. However, two pieces of evidence argue that IL-4 signaling is not required for the acquisition of Th2 locus demethylation during fetal life. First, our earlier study [9] found that the Th2 locus was demethylated in STAT6-deficient neonatal CD4+ cells to the same level as that seen in wild-type neonatal CD4+ cells. Second, we have recently found that CNS-1 is similarly hypomethylated in wildtype (Figure 3) and IL-4Rα-deficient fetal thymocytes (77.2±5.9% unmethylated). Therefore, the “rules” governing the epigenetic state of the Th2 locus may be different during fetal and adult life. Intriguingly, the window of developmental time in which the Th2 locus becomes demethylated within the fetal thymus overlaps with the interval in which imprinted genes become demethylated in primordial germ cells. Thus, it is possible that there may be shared mechanistic aspects. Of note, it has been recently described that active demethylation in the primordial germ cells is dependent on the enzyme activation-induced cytidine deaminase (AID) [34]. If AID is also involved in the demethylation of the Th2 locus, one clear prediction is that AID-deficient mice will show hypermethylation of the Th2 locus in fetal thymocytes.

One of the interesting observations in these data is the heterogeneity in the methylation patterns, particularly for CNS-1. For example, in day 1 CD4 SP thymocytes, clones fall into two distinct categories – either largely unmethylated or largely methylated (Fig. 1B). This could arise in several possible ways. First, there may be stochastically different responses to the same environmental signals within an otherwise homogeneous cell population. There is a precedent for this possibility. Guo et al [13] reported that naïve CD4+ lymph node cells cultured under identical Th2 conditions gave rise to clones with high or low frequencies of IL-4-producing cells. In the former case, CNS-1 was entirely unmethylated and, in the latter case, CNS-1 was heavily methylated. Second, the heterogeneity we see in CNS-1 may be a reflection of heterogeneity in the cell population analyzed. Some NKT cells express CD4 and could potentially have been included in our selected CD4+ population. However, that seems unlikely since thymic NKT cells are rare and do not appear until relatively late in mouse ontogeny, well after conventional T cells [35], [36], [37], [38], [39]. Similar arguments apply to the γδ lineage since CD4-expressing γδ+ cells are barely detectable in the neonatal thymus {Azuara, 1997 #2777}. An alternative possibility is that the CD4+ cells with the highly methylated versus unmethylated CNS-1 derive from different precursor populations. There is evidence from both the murine and avian systems that the embryonic thymus is seeded by discrete waves of precursor cells, with the last wave entering near the end of gestation [40], [41], [42], [43]. This last wave is thought to have more adult-like properties [44] and may be the primary source of neonatal CD4+ cells with an adult-like hypermethylated CNS-1. Thus, neonatal clones with fully methylated CNS-1 may represent those cells that will stably populate the periphery in adult animals.

We have shown that the CNS-1 region of the Th2 locus exists in a hypomethylated state in early life, in the absence of permissive acetylated or methylated histone marks. This is a surprising finding for two reasons. First, in developing adult Th2 effector cells, CNS-1 becomes marked with permissive modifications prior to DNA demethylation [27]. Second, as a general rule, unmethylated DNA is largely associated with acetylated histones [45]. Therefore, it appears that there may be a developmental window during which the Th2 locus has a unique epigenetic profile. Interestingly, in this regard, the Th2 locus resembles the global state attained by primordial germ cells at the endpoint of their reprogramming – i.e., the cells exist in an epigenetic ground state with genome wide demethylation and a lack of permissive histone marks [34], [46], [47].

The distinct epigenetic state of the Th2 locus in early life may serve to bias responses to the Th2 lineage without completely excluding the possibility of Th1 function. Neonatal life is a unique developmental phase during which organisms encounter a multitude of new antigens. Responding to many antigens simultaneously with highly inflammatory responses would likely lead to cytokine storms detrimental to developing organs. Therefore, it is probably advantageous to have immune responses set in a default anti-inflammatory Th2 mode. However, the hypomethylated state, by itself, is probably not sufficient for immediate, high-level Th2 cytokine production. It seems likely that favorable histone marks must also be acquired prior to extensive Th2 cytokine gene transcription by neonatal cells. We previously showed [48] that the most rapid IL-4 production is found within the population of neonatal lymph node CD4+ cells that has undergone homeostatic proliferation. This proliferation may be necessary for both the acquisition of favorable histone marks and the upregulation of the appropriate transcription factors. In fact, IL-4 production by the naïve (non-proliferated) neonatal population, although considerably faster than that by naïve adult cells, is delayed by an additional day. This delay may be important to allow the initiation of some Th1 function which could be essential, e.g., in cases of infection with pathogenic microorganisms. Therefore, we believe that hypomethylation of the Th2 locus in neonates acts to favor Th2 responses but not at the expense of completely eliminating Th1 responses.

## References

[1] Siegrist CA (2000) Vaccination in the neonatal period and early infancy. Int Rev Immunol 19: 195–219. 10763709 10.3109/08830180009088505

[2] Fadel S, Sarzotti M (2000) Cellular immune responses in neonates. Int Rev Immunol 19: 173–193. 10763708 10.3109/08830180009088504

[3] Adkins B (2000) Development of neonatal Th1/Th2 function. IntRevImmunol 19: 157–171. 10.3109/08830180009088503 10763707

[4] Prabhudas M, Adkins B, Gans H, King C, Levy O, et al (2011) Challenges in infant immunity: implications for responses to infection and vaccines. Nat Immunol 12: 189–194. 21321588 10.1038/ni0311-189

[5] Ribeiro-do-Couto LM, Boeije LC, Kroon JS, Hooibrink B, Breur-Vriesendorp BS, et al (2001) High IL-13 production by human neonatal T cells: neonate immune system regulator? Eur J Immunol 31: 3394–3402. 11745358 10.1002/1521-4141(200111)31:11<3394::aid-immu3394>3.0.co;2-b

[6] Webster RB, Rodriguez Y, Klimecki WT, Vercelli D (2007) The human IL-13 locus in neonatal CD4+ T cells is refractory to the acquisition of a repressive chromatin architecture. J Biol Chem 282: 700–709. 17090525 10.1074/jbc.M609501200

[7] Upham JW, Rate A, Rowe J, Kusel M, Sly PD, et al (2006) Dendritic cell immaturity during infancy restricts the capacity to express vaccine-specific T-cell memory. Infect Immun 74: 1106–1112. 16428758 10.1128/IAI.74.2.1106-1112.2006 PMC1360347

[8] Prescott SL, Macaubas C, Holt BJ, Smallacombe TB, Loh R, et al (1998) Transplacental priming of the human immune system to environmental allergens: universal skewing of initial T cell responses toward the Th2 cytokine profile. J Immunol 160: 4730–4737. 9590218

[9] Rose S, Lichtenheld M, Foote M, Adkins B (2007) Murine neonatal CD4+ cells are poised for rapid Th2 effector-like function. J Immunol 178: 2667–2678. 17312108 10.4049/jimmunol.178.5.2667 PMC2112939

[10] Ansel KM, Djuretic I, Tanasa B, Rao A (2006) Regulation of Th2 differentiation and Il4 locus accessibility. Annu Rev Immunol 24: 607–656. 16551261 10.1146/annurev.immunol.23.021704.115821

[11] Lee GR, Fields PE, Flavell RA (2001) Regulation of IL-4 gene expression by distal regulatory elements and GATA-3 at the chromatin level. Immunity 14: 447–459. 11336690 10.1016/s1074-7613(01)00125-x

[12] Loots GG, Locksley RM, Blankespoor CM, Wang ZE, Miller W, et al (2000) Identification of a coordinate regulator of interleukins 4, 13, and 5 by cross-species sequence comparisons. Science 288: 136–140. 10753117 10.1126/science.288.5463.136

[13] Guo L, Hu-Li J, Zhu J, Watson CJ, Difilippantonio MJ, et al (2002) In TH2 cells the Il4 gene has a series of accessibility states associated with distinctive probabilities of IL-4 production. Proc Natl Acad Sci U S A 99: 10623–10628. 12149469 10.1073/pnas.162360199 PMC124993

[14] Aoki K, Sato N, Yamaguchi A, Kaminuma O, Hosozawa T, et al (2009) Regulation of DNA demethylation during maturation of CD4+ naive T cells by the conserved noncoding sequence 1. J Immunol 182: 7698–7707. 19494294 10.4049/jimmunol.0801643

[15] Makar KW, Perez-Melgosa M, Shnyreva M, Weaver WM, Fitzpatrick DR, et al (2003) Active recruitment of DNA methyltransferases regulates interleukin 4 in thymocytes and T cells. Nat Immunol 4: 1183–1190. 14595437 10.1038/ni1004

[16] Calvert JE, Kim MF, Gathings WE, Cooper MD (1983) Differentiation of B lineage cells from liver of neonatal mice: generation of immunoglobulin isotype diversity in vitro. J Immunol 131: 1693–1697. 6604750

[17] Ikawa T, Masuda K, Lu M, Minato N, Katsura Y, et al (2004) Identification of the earliest prethymic T-cell progenitors in murine fetal blood. Blood 103: 530–537. 14512296 10.1182/blood-2003-06-1797

[18] Kawamoto H, Ikawa T, Ohmura K, Fujimoto S, Katsura Y (2000) T cell progenitors emerge earlier than B cell progenitors in the murine fetal liver. Immunity 12: 441–450. 10795742 10.1016/s1074-7613(00)80196-x

[19] Schmitt TM, Zuniga-Pflucker JC (2002) Induction of T cell development from hematopoietic progenitor cells by delta-like-1 in vitro. Immunity 17: 749–756. 12479821 10.1016/s1074-7613(02)00474-0

[20] Yoshimoto M, Porayette P, Glosson NL, Conway SJ, Carlesso N, et al (2012) Autonomous murine T-cell progenitor production in the extra-embryonic yolk sac before HSC emergence. Blood 119: 5706–5714. 22431573 10.1182/blood-2011-12-397489 PMC3382930

[21] Adkins B (2003) Peripheral CD4(+) Lymphocytes Derived from Fetal versus Adult Thymic Precursors Differ Phenotypically and Functionally. J Immunol 171: 5157–5164. 14607915 10.4049/jimmunol.171.10.5157

[22] Adkins B, Hamilton K (1994) Developmental ages of the thymic epithelium and of the T cell precursors together determine the proportions of peripheral CD4+ cells. J Immunol 153: 5359–5365. 7989742

[23] Tykocinski LO, Hajkova P, Chang HD, Stamm T, Sozeri O, et al (2005) A critical control element for interleukin-4 memory expression in T helper lymphocytes. J Biol Chem 280: 28177–28185. 15941711 10.1074/jbc.M502038200

[24] Fields PE, Lee GR, Kim ST, Bartsevich VV, Flavell RA (2004) Th2-specific chromatin remodeling and enhancer activity in the Th2 cytokine locus control region. Immunity 21: 865–876. 15589174 10.1016/j.immuni.2004.10.015

[25] Schoenborn JR, Dorschner MO, Sekimata M, Santer DM, Shnyreva M, et al (2007) Comprehensive epigenetic profiling identifies multiple distal regulatory elements directing transcription of the gene encoding interferon-gamma. Nat Immunol 8: 732–742. 17546033 10.1038/ni1474 PMC2144744

[26] Floess S, Freyer J, Siewert C, Baron U, Olek S, et al (2007) Epigenetic control of the foxp3 locus in regulatory T cells. PLoS Biol 5: e38. 17298177 10.1371/journal.pbio.0050038 PMC1783672

[27] Wilson CB, Rowell E, Sekimata M (2009) Epigenetic control of T-helper-cell differentiation. Nat Rev Immunol 9: 91–105. 19151746 10.1038/nri2487

[28] Grogan JL, Wang ZE, Stanley S, Harmon B, Loots GG, et al (2003) Basal chromatin modification at the IL-4 gene in helper T cells. J Immunol 171: 6672–6679. 14662870 10.4049/jimmunol.171.12.6672

[29] Bhandoola A, von Boehmer H, Petrie HT, Zuniga-Pflucker JC (2007) Commitment and developmental potential of extrathymic and intrathymic T cell precursors: plenty to choose from. Immunity 26: 678–689. 17582341 10.1016/j.immuni.2007.05.009

[30] Rhodes KE, Gekas C, Wang Y, Lux CT, Francis CS, et al (2008) The emergence of hematopoietic stem cells is initiated in the placental vasculature in the absence of circulation. Cell Stem Cell 2: 252–263. 18371450 10.1016/j.stem.2008.01.001 PMC2888040

[31] Martino D, Maksimovic J, Joo JH, Prescott SL, Saffery R (2012) Genome-scale profiling reveals a subset of genes regulated by DNA methylation that program somatic T-cell phenotypes in humans. Genes Immun. 10.1038/gene.2012.7 22495533

[32] Wilson CB, Makar KW, Shnyreva M, Fitzpatrick DR (2005) DNA methylation and the expanding epigenetics of T cell lineage commitment. Semin Immunol 17: 105–119. 15737572 10.1016/j.smim.2005.01.005

[33] Lee DU, Agarwal S, Rao A (2002) Th2 lineage commitment and efficient IL-4 production involves extended demethylation of the IL-4 gene. Immunity 16: 649–660. 12049717 10.1016/s1074-7613(02)00314-x

[34] Popp C, Dean W, Feng S, Cokus SJ, Andrews S, et al (2010) Genome-wide erasure of DNA methylation in mouse primordial germ cells is affected by AID deficiency. Nature 463: 1101–1105. 20098412 10.1038/nature08829 PMC2965733

[35] Bendelac A (1995) Mouse NK1+ T cells. Curr Opin Immunol 7: 367–374. 7546402 10.1016/0952-7915(95)80112-x

[36] Benlagha K, Kyin T, Beavis A, Teyton L, Bendelac A (2002) A thymic precursor to the NK T cell lineage. Science 296: 553–555. 11968185 10.1126/science.1069017

[37] Hammond K, Cain W, van Driel I, Godfrey D (1998) Three day neonatal thymectomy selectively depletes NK1.1+ T cells. Int Immunol 10: 1491–1499. 9796916 10.1093/intimm/10.10.1491

[38] MacDonald HR (1995) NK1.1+ T cell receptor-alpha/beta+ cells: new clues to their origin, specificity, and function. J Exp Med 182: 633–638. 7650474 10.1084/jem.182.3.633 PMC2192172

[39] Pellicci DG, Hammond KJ, Uldrich AP, Baxter AG, Smyth MJ, et al (2002) A natural killer T (NKT) cell developmental pathway iInvolving a thymus-dependent NK1.1(-)CD4(+) CD1d-dependent precursor stage. J Exp Med 195: 835–844. 11927628 10.1084/jem.20011544 PMC2193721

[40] Jotereau F, Heuze F, Salomon-Vie V, Gascan H (1987) Cell kinetics in the fetal mouse thymus: precursor cell input, proliferation, and emigration. J Immunol 138: 1026–1030. 2879866

[41] Coltey M, Bucy RP, Chen CH, Cihak J, Losch U, et al (1989) Analysis of the first two waves of thymus homing stem cells and their T cell progeny in chick-quail chimeras. J Exp Med 170: 543–557. 2666562 10.1084/jem.170.2.543 PMC2189409

[42] Jotereau FV, Le Douarin NM (1982) Demonstration of a cyclic renewal of the lymphocyte precursor cells in the quail thymus during embryonic and perinatal life. J Immunol 129: 1869–1877. 7119436

[43] Coltey M, Jotereau FV, Le Douarin NM (1987) Evidence for a cyclic renewal of lymphocyte precursor cells in the embryonic chick thymus. Cell Differ 22: 71–82. 3690675 10.1016/0045-6039(87)90414-3

[44] Mold JE, Venkatasubrahmanyam S, Burt TD, Michaelsson J, Rivera JM, et al (2010) Fetal and adult hematopoietic stem cells give rise to distinct T cell lineages in humans. Science 330: 1695–1699. 21164017 10.1126/science.1196509 PMC3276679

[45] Cedar H, Bergman Y (2009) Linking DNA methylation and histone modification: patterns and paradigms. Nat Rev Genet 10: 295–304. 19308066 10.1038/nrg2540

[46] Hajkova P, Ancelin K, Waldmann T, Lacoste N, Lange UC, et al (2008) Chromatin dynamics during epigenetic reprogramming in the mouse germ line. Nature 452: 877–881. 18354397 10.1038/nature06714 PMC3847605

[47] Seki Y, Yamaji M, Yabuta Y, Sano M, Shigeta M, et al (2007) Cellular dynamics associated with the genome-wide epigenetic reprogramming in migrating primordial germ cells in mice. Development 134: 2627–2638. 17567665 10.1242/dev.005611

[48] Adkins B, Guevara P, Rose S (2006) Thymic and extrathymic contributions to T helper cell function in murine neonates. Heamatologica reports 2: 9–13. PMC2948465 20890454

